# A Robust Skeletonization Method for High-Density Fringe Patterns in Holographic Interferometry Based on Parametric Modeling and Strip Integration

**DOI:** 10.3390/jimaging12020054

**Published:** 2026-01-24

**Authors:** Sergey Lychev, Alexander Digilov

**Affiliations:** Ishlinsky Institute for Problems in Mechanics RAS, 119526 Moscow, Russia; digilov@ipmnet.ru

**Keywords:** holographic interferometry, fringe skeletonization, speckle noise, parametric modeling, strip integration, displacement field reconstruction

## Abstract

Accurate displacement field measurement by holographic interferometry requires robust analysis of high-density fringe patterns, which is hindered by speckle noise inherent in any interferogram, no matter how perfect. Conventional skeletonization methods, such as edge detection algorithms and active contour models, often fail under these conditions, producing fragmented and unreliable fringe contours. This paper presents a novel skeletonization procedure that simultaneously addresses three fundamental challenges: (1) topology preservation—by representing the fringe family within a physics-informed, finite-dimensional parametric subspace (e.g., Fourier-based contours), ensuring global smoothness, connectivity, and correct nesting of each fringe; (2) extreme noise robustness—through a robust strip integration functional that replaces noisy point sampling with Gaussian-weighted intensity averaging across a narrow strip, effectively suppressing speckle while yielding a smooth objective function suitable for gradient-based optimization; and (3) sub-pixel accuracy without phase extraction—leveraging continuous bicubic interpolation within a recursive quasi-optimization framework that exploits fringe similarity for precise and stable contour localization. The method’s performance is quantitatively validated on synthetic interferograms with controlled noise, demonstrating significantly lower error compared to baseline techniques. Practical utility is confirmed by successful processing of a real interferogram of a bent plate containing over 100 fringes, enabling precise displacement field reconstruction that closely matches independent theoretical modeling. The proposed procedure provides a reliable tool for processing challenging interferograms where traditional methods fail to deliver satisfactory results.

## 1. Introduction

Holographic interferometry is a cornerstone optical technique for full-field, non-contact measurement of displacements and deformations in experimental mechanics [1]. Its high sensitivity, enabling resolution of displacements on the order of the light wavelength, makes it invaluable for studying complex deformation phenomena. The accuracy of the method is intrinsically connected with the precision with which the resulting interference fringe patterns can be analyzed [2]. In principle, higher measurement resolution is achieved by analyzing interferograms with a high density of fringes. However, in modern practice, the outcome becomes critically dependent on the chosen digital image processing algorithm, as the manual detection of hundreds of fringes is unreliable [3].

The primary obstacle to automation is the presence of speckle noise—an unavoidable granular interference pattern generated by the random scattering of coherent light from surface micro-roughness, which is intrinsic to any physical hologram [4]. While post-processing filters (e.g., median or Gaussian) can reduce speckle visibility in a digital image, they require a delicate approach. Intensive smoothing can introduce systematic biases that shift the fringe locations, a particularly detrimental effect for high-density patterns where fine details are essential [5]. Consequently, the development of robust, noise-resistant skeletonization algorithms that can dispense with significant pre-filtering remains an actual problem in optical metrology.

A variety of general-purpose image-processing techniques have been applied to fringe identification, each with intrinsic limitations for the high-density, high-noise regime we address:

- Phase-based techniques (phase-shifting/unwrapping) (e.g., [6,7,8]) achieve high precision but operate in a different paradigm: they typically analyze *projected* fringe patterns for 3D shape measurement and require *multiple phase-stepped frames*, making them unsuitable for single-shot analysis of intrinsic holographic interference patterns encoding nanoscale displacements.
- Edge and ridge detection algorithms (e.g., Marr-Hildreth [9], Canny [10], Shen–Castan [11]) can localize fringes under moderate noise. However, their output is inherently a *fragmented map of pixels or short edge segments*. For dense patterns corrupted by strong speckle noise, this map becomes a scattered point cloud. The subsequent critical step—reassembling these fragments into topologically correct, smooth, and closed analytical curves—is a complex and often unreliable post-processing challenge.
- Active contour models (snakes) and their variants (e.g., Gradient Vector Flow snakes [12,13]) formulate fringe extraction as an energy minimization problem, offering a continuous curve representation [14,15,16,17,18]. Despite their flexibility, they exhibit two key limitations for our target conditions: (1) high sensitivity to initial position, often causing convergence to an adjacent fringe or leakage in high-density patterns; and (2) an inherent tension/rigidity regularization that acts as a low-pass filter, potentially oversmoothing the contour and introducing systematic localization bias in the presence of high-frequency speckle noise.
- Learning-based approaches (e.g., [19,20,21]) promise robustness but require extensive, high-quality labeled datasets, which are scarce for specialized tasks like analyzing specific deformation patterns under extreme noise conditions typical in experimental mechanics.

To overcome these limitations, we propose a novel skeletonization procedure that strategically integrates physical prior knowledge with a noise-robust numerical core. Our contribution is threefold:

1. Physics-informed parametric modeling: We constrain the search for fringes to a purpose-built, finite-dimensional functional subspace $A_{\theta }$, which is defined by specific parametric curves (e.g., trigonometric polynomials or splines). This subspace is constructed in such a way as to endow its elements with the required properties, such as smoothness, closure, and specific shape, guaranteeing that the identified fringes are physically plausible, connected curves.
2. A robust strip integration functional with local smoothing: For fringe localization we consider the specific functional in the form of a Gaussian-weighted integral of intensity over a narrow strip surrounding the candidate curve. This formulation allows for the use of efficient gradient-based optimization techniques. To compute this functional, a continuously differentiable intensity field $\tilde{I}\left(x,y\right)$ is first obtained by local bicubic interpolation, described in Section 3.1. The resulting functional $J^{s}\left(\theta \right)$ is smooth with respect to the curve parameters θ, ensuring stable convergence.
3. A recursive quasi-optimization algorithm: Exploiting the geometric similarity of adjacent fringes, the identification process proceeds recursively outward (or inward). The optimal parameters for an identified fringe seed the initial guess for the next, via a simple scaling transformation (*quasi-optimization*), followed by a full local refinement of all parameters. This strategy dramatically improves computational efficiency and reliability.

The performance of the proposed method is carefully validated. First, using synthetic interferograms with precisely controlled geometry and additive-multiplicative speckle noise, we demonstrate its acceptable accuracy and robustness compared to baseline methods. The error is estimated using two metrics: the Euclidean norm ($L^{2}$-norm) of the difference and its maximal absolute value ($L^{\infty }$-norm). Second, we apply the algorithm to a real, challenging interferogram from a bending experiment on a square plate, containing over 100 fringes. The algorithm successfully extracts the complete skeleton, enabling an accurate reconstruction of the displacement field w(x,y). This result is shown to be in good agreement with an independent analytical solution, obtained in [22], confirming the practical utility and accuracy of the entire procedure.

The remainder of this paper is organized as follows: Section 2 details the proposed skeletonization procedure. Section 3 describes key implementation aspects. Section 4 presents the quantitative validation on synthetic data. Section 5 showcases the application to real high-density interferograms. Finally, Section 6 discusses the results and outlines future work.

## 2. The Proposed Skeletonization Method

### 2.1. Physical Origins of Imperfections in Interferograms

To motivate the design choices of the proposed algorithm, it is essential to understand the physical nature of the imperfections present in a raw interferogram. These imperfections, which corrupt the ideal sinusoidal fringe pattern, arise from the optical setup imperfections and the nature of coherent light scattering. They can be categorized by their spatial scale, necessitating different corrective strategies in the digital processing procedure.

In a typical off-axis holographic setup (e.g., the Leith–Upatnieks scheme [2]), the recorded intensity results from the interference of a reference plane wave $E_{r}\left(r\right)$ and an object wave $E_{o}\left(r\right)$ reflected from the specimen surface. In complex notation, these waves are as follows:

$$\begin{array}{cc} E_{r}\left(r\right) & =A_{r}e_{r}\;e^{ik_{r}\cdot r},\\ E_{o}\left(r\right) & =A_{o}\left(x,y\right)\;J\left(x,y\right)\;e_{o}\;e^{ik_{o}\cdot r}, \end{array}$$

where $A_{r}$ and $A_{o}\left(x,y\right)$ are the scalar amplitudes, $e_{r}$ and $e_{o}$ are the unit polarization vectors of the reference and initial object waves (directly from the laser source), $k_{r}$ and $k_{o}$ are the corresponding wave vectors, and J(x,y) is the spatially varying Jones matrix accounting for polarization changes upon reflection from the deformed surface.

The interference of the wavefronts from the specimen’s reference and deformed states yields an intensity pattern of the following form [4,23]:

(1) $$I_{\mathrm{obs}}\left(x,y\right)\propto I_{0}\left(x,y\right)\left[1+V\left(x,y\right)\cos\;\left(\Delta \phi (x,y)\right)\right]R\left(x,y\right)+“\mathrm{noise}”.$$

Here, Δϕ(x,y)=(4π/λ)w(x,y) is the phase difference directly proportional to the deformable object out-of-plane displacement w(x,y), $V\left(x,y\right)\propto |\left(J_{1}e_{o}\right)\cdot {\left(J_{2}e_{o}\right)}^{*}|$ stands for the polarization-induced visibility term, and R(x,y) is the contrast variation, induced by coherent noise. A transition between consecutive dark (or bright) fringes corresponds to a displacement increment of λ/2.

The terms $I_{0}\left(x,y\right)$, V(x,y) and R(x,y) describe large- to medium-scale corruptions:

- **Non-uniform background intensity $I_{0}\left(x,y\right)$:** This is caused by uneven illumination, varying surface reflectivity, and polarization effects ($J_{1}\neq J_{2}$). This leads to slow intensity variations across the image.
- **Visibility term and contrast variation V(x,y),R(x,y):** The coherence noisy factor R(x,y) (dependent on laser temporal/spectral properties and surface roughness) modulates the overall fringe contrast. The visibility term V(x,y) induced by polarization and speckle decorrelation can further reduce contrast, even to zero.
- **Geometric distortion:** The non-coaxiality of reference and object beams in off-axis schemes, combined with lens imperfections, introduces a projective distortion between the object plane and the image sensor.

These low-frequency imperfections are corrected in the **pre-processing stage** (Section 3) via geometric unwarping and local intensity equalization.

The speckle-dependent terms in (1) present the most significant challenge. It is a fine-grained, high-frequency random pattern resulting from two physically unavoidable phenomena: (i) the finite spectral bandwidth of the laser source, and (ii) the random interference of waves scattered by surface micro-roughness. Unlike Gaussian additive noise, speckle is signal-dependent and exhibits characteristic correlation lengths ($\rho_{\mathrm{speckle}}$). Its spatial frequency content often overlaps with that of the high-density fringes themselves, making linear filtering unsuitable as it erodes fringe edges and introduces systematic localization errors.

This multi-scale analysis dictates our algorithmic strategy: large-scale distortions are removed by explicit pre-processing, while the fine-scale speckle noise is addressed not by filtering but by the main idea of the proposed method—the **parametric modeling** (Section 2.2) and the **strip integration functional** (Section 2.4)—which provides robustness without distorting the signal.

### 2.2. Parametric Modeling and Problem Formulation

The goal of skeletonization is to extract a family of planar curves $\Gamma ={\left\{\gamma_{i}\right\}}_{i=1}^{N}$ that represent the level sets (fringes) of the phase map Δϕ(x,y) from a discretely sampled intensity image *I*. A fundamental premise of our approach is to treat fringes as *continuous geometric entities* (curves) rather than collections of disconnected pixels. Consequently, the search for an optimal curve γ must be performed within a suitable functional space [24].

Let $\Omega \subset R^{2}$ denote the rectangular image domain. We consider the space of admissible curves V to be a set of rectifiable, closed Jordan curves contained in Ω. For any such curve γ∈V, we define a functional as the normalized integral of the image intensity over its support:

$$J\left(\gamma \right)=\frac{1}{\mu (\gamma )}\int_{\gamma }I\;d\mu ,$$

where μ is an appropriate measure on γ. In the simplest case, where the support is the one-dimensional curve itself, μ is the arc-length measure (dμ=ds), and the normalization factor μ(γ) is the total curve length L(γ). This yields the *line-integral functional*:

$$J^{l}\left(\gamma \right)=\frac{1}{L(\gamma )}\oint_{\gamma }I\;ds.$$

The choice of measure and its domain will be generalized in Section 2.4 to define more robust functionals. The core optimization problem is to find curves within a constrained set that extremize this functional. For an ideal, normalized interferogram where the low-frequency imperfections from (1) are absent ($I_{0}\equiv \mathrm{const}.$, V≡1,R≡1) and speckle noise is removed, the intensity is proportional to 1+cos(Δϕ(x,y)). The bright fringes (intensity maxima) correspond to curves that maximize $J^{l}$, while dark fringes (minima) correspond to its minimizers. In a real, noisy image, the functional $J^{l}$ must be extremized despite the corruptions described in Section 2.1.

However, operating directly in the infinite-dimensional space V is intractable and ignores the crucial physical prior: the expected fringe family possesses a specific global structure. Instead of regularizing the problem within V (as active contours do), we *constrain* the solution a priori to a finite-dimensional subspace $A_{\theta }\subset V$. This subspace is defined by a parametric model:

(2) $$A_{\theta }=\left\{\gamma \left(\theta \right)|\theta \in \Theta \subset R^{m}\right\},$$

where $\theta =(\theta_{1},\ldots ,\theta_{m})$ is a vector of parameters, and the mapping θ↦γ(θ) is continuously differentiable. The choice of the model $A_{\theta }$ takes into account our a priori knowledge about the deformation. For instance, for the bending of a centrally loaded, clamped plate, the fringes are expected to be nested, closed, and quasi-concentric curves, which can be effectively modeled by a family of perturbed circles or ellipses (see Section 3.4).

By restricting the search to $A_{\theta }$, the infinite-dimensional variational problem of extremizing $J^{l}$ over V reduces to a finite-dimensional optimization problem over the parameter space Θ:

$$\theta^{*}=\underset{\theta \in \Theta }{arg extr}J^{l}\left(\gamma \left(\theta \right)\right).$$

This is the core conceptual shift from other methods: the smoothness, connectivity, and global shape of the solution are *built into* the search space $A_{\theta }$, rather than being enforced via penalty terms during optimization.

### 2.3. Discretization and Coordinate System

The image data *I* are provided as a discrete matrix I[r,c] of size $r_{\max}\times c_{\max}$, where *r* and *c* are integer row and column indices. To connect the continuous formulation with the discrete data, we establish a fixed coordinate system. We assume a unit pixel pitch and place the origin of the Cartesian coordinates (x,y) at the center of a chosen reference pixel with indices $\left(r_{0},c_{0}\right)$. The correspondence between a pixel P[[r,c]] and the coordinates of its center $\left(x_{P},y_{P}\right)$ is then given as follows:

(3) $$\left(x,y\in P\left[\;\left[r,c\right]\;\right]\right)\Leftrightarrow \left(r=r_{0}-\left\lfloor y+1/2\right\rfloor ,c=c_{0}+\left\lfloor x+1/2\right\rfloor \right).$$

**Figure 1:**
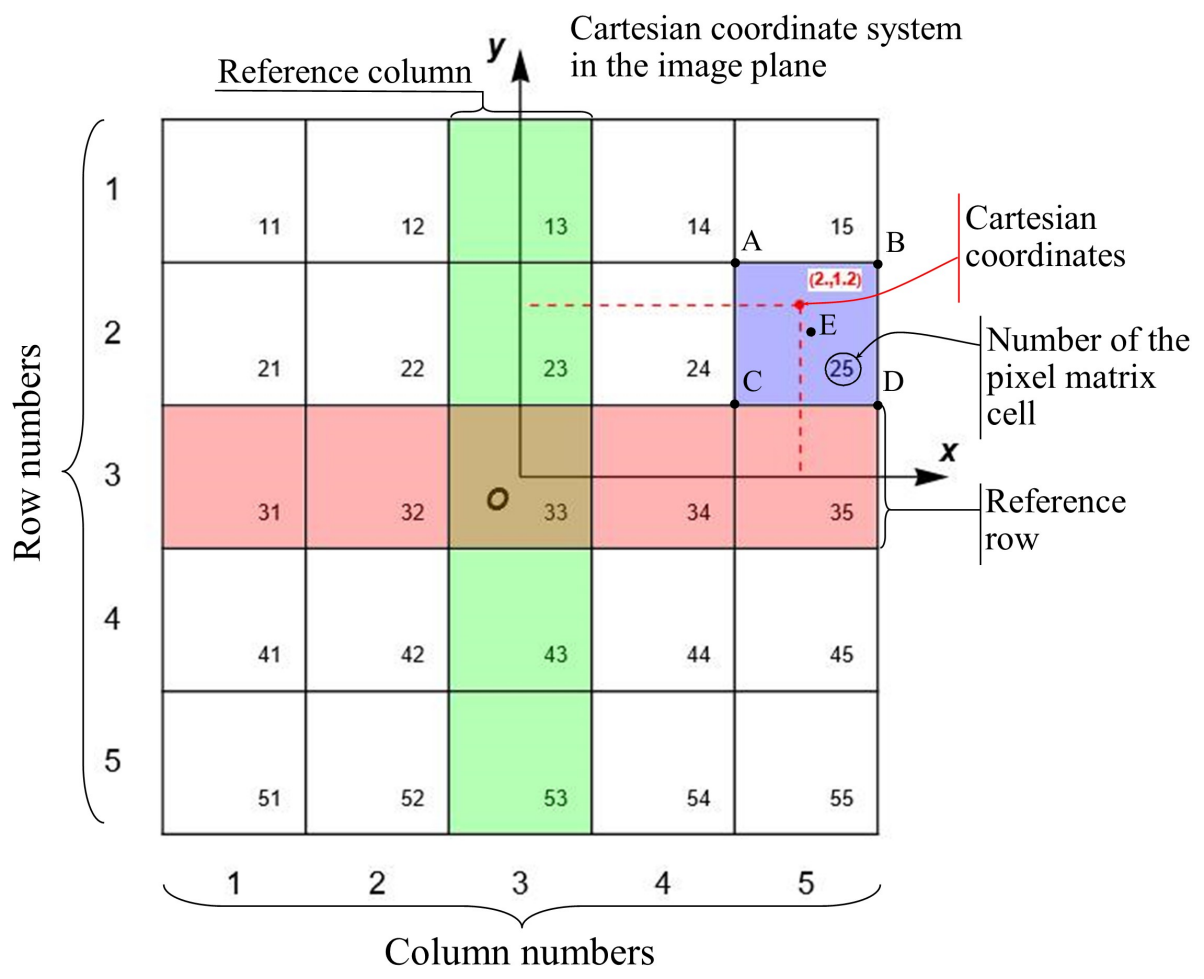
Relationship between pixel matrix indices (r,c) and the continuous Cartesian coordinate system (x,y). The grid represents the pixel centers. The origin (x,y)=(0,0) is fixed at the center of the reference pixel with indices $\left(r_{0},c_{0}\right)=\left(3,3\right)$. The coordinates of point *E* (the center of pixel (2,5)) are $\left(x_{E},y_{E}\right)=\left(1,2\right)$, following Equation (3). The vertices A,B,C,D denote the corners of a pixel, located at ±0.5 offsets from its center.

This mapping is illustrated in Figure 1.

### 2.4. Variants of the Functional

The core of the skeletonization method is the optimization of a functional that measures how well a candidate curve aligns with a fringe. We trace its development from a naive discrete form to the final robust, continuous formulation.

#### 2.4.1. Point-Sampling Along the Curve

The most direct approach is to rasterize the curve γ(θ) and sum the intensities of its pixels:

$$J_{\mathrm{point}}^{l}\left(\theta \right)=\frac{1}{N(\theta )}\;\sum_{R(\gamma (\theta ))}\;I\left[r,c\right],$$

where R is the set of pixels that rasterize the curve, and N(θ)=|R| is their number (an integer measure of curve length). This functional is *discontinuous* with respect to θ, which means that a small parameter change can alter the set R, causing a jump in the sum. This discontinuity precludes the use of gradient-based optimization.

#### 2.4.2. Line-Integration with Interpolation

To obtain a smooth functional, we first construct a $C^{1}$-continuous intensity field $\tilde{I}\left(x,y\right)$ via bicubic interpolation (detailed in Section 3.1). Replacing the discrete sum with a normalized line integral yields the following:

(4) $$J^{l}\left(\theta \right)=\frac{1}{L(\theta )}\oint_{\gamma (\theta )}\tilde{I}\left(x,y\right)\;ds,$$

where L(θ) is the curve length. $J^{l}\left(\theta \right)$ is now continuously differentiable, enabling gradient-based optimization. However, it remains sensitive to speckle noise, as it samples intensity only along an infinitesimally thin path.

#### 2.4.3. Point-Sampling over a Strip

To improve robustness, we consider a strip S(γ,δ) of width 2δ around the curve. A discrete, robust functional can be defined by summing pixel intensities within this strip, weighted by their approximate Gaussian distance to the curve:

$$J_{\mathrm{point}}^{s}\left(\theta \right)=\frac{1}{N(\theta )}\sum_{R_{S}}G\;\left(\frac{d_{rc}}{\sigma }\right)I\left[r,c\right],$$

where $R_{S}$ is the set of pixels whose centers lie within the strip S, $d_{rc}$ is the minimal distance from the pixel center to the curve, and $G\left(u\right)=\exp\left(-u^{2}/2\right)$. This choice of weight function is motivated by the statistical nature of speckle noise nature. The functional $J_{\mathrm{point}}^{s}$ averages over many pixels, reducing the impact of speckles. However, it shares the discontinuity flaw of $J_{\mathrm{point}}^{l}$ due to the discrete pixel set $R_{S}$.

#### 2.4.4. Strip-Integration with Interpolation

The final, optimal form combines the robustness of strip averaging with the smoothness of continuous integration. We define the *strip integration functional* as the normalized, weighted area integral over the continuous strip:

(5) $$J^{s}\left(\theta \right)=\frac{\underset{S(\gamma (\theta ),\;\delta )}{\;\int \int \;}\;\;\;\;G\;\left(\frac{d(x,\gamma )}{\sigma }\right)\;\tilde{I}\left(x,y\right)\;dA}{\underset{S(\gamma (\theta ),\;\delta )}{\;\int \int \;}\;\;\;\;dA},$$

where d(x,γ) is the minimal Euclidean distance between x and the curve. The functional, $J^{s}$, is both robust to noise (due to area averaging) and continuously differentiable in θ (due to the smoothness of $\tilde{I}$, *G*, and the parametric curve). It is the functional used in our final algorithm.

## 3. Numerical Implementation

The evaluation of $J^{l}$ and $J^{s}$ requires efficient numerical techniques for interpolation and integration.

### 3.1. Bicubic Interpolation Scheme

To obtain the $C^{1}$ intensity field $\tilde{I}\left(x,y\right)$ from the pixel matrix I[r,c], we employ a compact bicubic formula. For a point (x,y) in a grid cell with corners at pixel centers, the interpolation is as follows:

$$\tilde{I}\left(x,y\right)=\left[\begin{array}{cccc} 1 & \Delta (x) & \Delta {\left(x\right)}^{2} & \Delta {\left(x\right)}^{3} \end{array}\right]\;M^{T}\;D\left(i\left(y\right),j\left(x\right)\right)\;M\;\left[\begin{array}{c} 1\\ \Delta (y)\\ \Delta {\left(y\right)}^{2}\\ \Delta {\left(y\right)}^{3} \end{array}\right],$$

where $\Delta \left(x\right)=x+\frac{1}{2}-\left\lfloor x+\frac{1}{2}\right\rfloor$, $\Delta \left(y\right)=y+\frac{1}{2}-\left\lfloor y+\frac{1}{2}\right\rfloor$ are local coordinates within a cell of the interpolation grid. The integer-valued indices i(y) and j(x), which locate the pixel, are functions of the coordinates:

(6) $$i\left(y\right)=r_{0}-\left\lfloor y+\frac{1}{2}\right\rfloor ,\;j\left(x\right)=c_{0}+\left\lfloor x+\frac{1}{2}\right\rfloor .$$

The constant matrix M and the matrix D(i,j) (now with i,j understood as the outputs of (6)) are as follows:

$$M=\left(\begin{array}{cccc} 1 & 0 & -3 & 2\\ 0 & 0 & 3 & -2\\ 0 & 1 & -2 & 1\\ 0 & 0 & -1 & 1 \end{array}\right),\;D\left(i,j\right)=\left(\begin{array}{cccc} f_{ij}^{00} & f_{ij}^{01} & h_{ij}^{00} & h_{ij}^{01}\\ f_{ij}^{10} & f_{ij}^{11} & h_{ij}^{10} & h_{ij}^{11}\\ g_{ij}^{00} & g_{ij}^{01} & q_{ij}^{00} & q_{ij}^{01}\\ g_{ij}^{10} & g_{ij}^{11} & q_{ij}^{10} & q_{ij}^{11} \end{array}\right).$$

**Figure 2:**
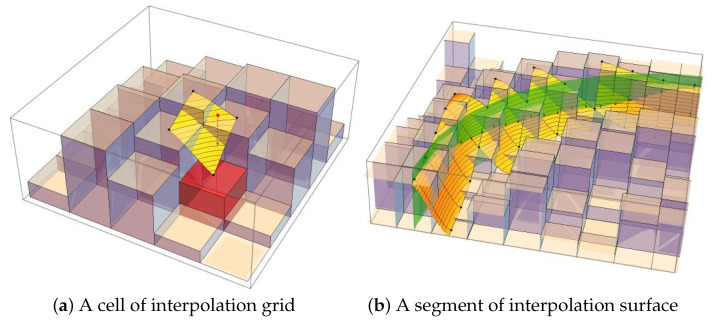
Bicubic interpolation.

The sixteen elements of D are finite-difference approximations of the intensity and its derivatives at the four cell corners, computed from the 4×4 neighborhood of the pixel (i,j):

$$\begin{array}{cc} f_{ij}^{pk} & =I[i-k,\;j+p],\\ g_{ij}^{pk} & =\frac{1}{2}\left(I[i-k,\;j+p+1]-I[i-k,\;j+p-1]\right),\\ h_{ij}^{pk} & =\frac{1}{2}\left(I[i-k-1,\;j+p]-I[i-k+1,\;j+p]\right),\\ q_{ij}^{pk} & =\frac{1}{4}(I\left[i-k-1,\;j+p+1\right]-I\left[i-k+1,\;j+p+1\right]\\ \; & \;\;\;\;\;\;-I[i-k-1,\;j+p-1]+I[i-k+1,\;j+p-1]), \end{array}$$

for p,k∈{0,1}. This scheme guarantees that $\tilde{I}\left(x,y\right)$ is a $C^{1}$ function across cell boundaries, providing the smoothness required for stable gradient computation. An example of interpolation is shown in Figure 2.

### 3.2. Numerical Quadrature

The line integral (4) is approximated by discretizing the curve into *n* segments. For a parametric curve (x(φ),y(φ)), φ∈[0,2π], we use the trapezoidal rule:

$$J^{l}\left(\theta \right)\approx \frac{\sum_{k=1}^{n}\left(\tilde{I}\left(x_{k},y_{k}\right)+\tilde{I}\left(x_{k-1},y_{k-1}\right)\right)\sqrt{{\left(x_{k}-x_{k-1}\right)}^{2}+{\left(y_{k}-y_{k-1}\right)}^{2}}}{2\sum_{k=1}^{n}\sqrt{{\left(x_{k}-x_{k-1}\right)}^{2}+{\left(y_{k}-y_{k-1}\right)}^{2}}},$$

where $\left(x_{k},y_{k}\right)=\left(x\left(\varphi_{k}\right)+1/2,y\left(\varphi_{k}\right)-1/2\right)$. The discretization step Δφ is chosen adaptively based on the curve’s local curvature to ensure at least one sample per intersected pixel:

$$\Delta ={\left({\dot{x}}^{2}+{\dot{y}}^{2}\right)}^{-1/2}.$$

In case of strip integration, the denominator of (5) can be computed more convenient analytically:

$$\underset{S(\gamma (\theta ),\;\delta )}{\;\int \int \;}\;\;\;\;dA=\int_{\gamma }\;\int_{-\delta }^{\delta }\;\;J\;d\xi \;d\varphi ,$$

using the expansion for the Jacobian determinant, *J*, of the coordinate transformation from (φ,ξ) (curve parameter and normal offset) to (x,y):

$$J=\left|\begin{array}{cc} \dot{x}-\xi \left(\ddot{y}\Delta +\dot{y}\dot{\Delta }\right)\; & \dot{y}+\xi \left(\ddot{x}\Delta +\dot{x}\dot{\Delta }\right)\\ -\dot{y}\Delta \; & \dot{x}\Delta \end{array}\right|=\frac{1}{\Delta }-\xi \left(\dot{x}\ddot{y}-\ddot{x}\dot{y}\right)\Delta^{2}.$$

Substituting this relation into (5) brings it to a form similar to (4):

$$J^{s}\left(\theta \right)=\frac{2\delta }{L(\theta )}\underset{S(\gamma (\theta ),\;\delta )}{\;\int \int \;}\;\;\;\;G\;\left(\frac{d(x,\gamma )}{\sigma }\right)\;\tilde{I}\left(x,y\right)\;dA,$$

This integral is approximated by summing contributions from triangular prisms formed by adjacent sample points:

$$J^{s}\left(\theta \right)\approx \frac{2\delta }{L(\theta )}\sum_{k=1}^{n-1}\sum_{p=-P}^{P-1}\left[V\left(x_{k}^{p},x_{k}^{p+1},x_{k+1}^{p}\right)+V\left(x_{k}^{p+1},x_{k+1}^{p+1},x_{k+1}^{p}\right)\right],$$

where $x_{k}^{p}=x_{k}+\left(p\delta /P\right)\;n_{k}$ are the coordinates of a point along the normal $n_{k}$ at $x_{k}$, and V(A,B,C) is the volume of a triangular prism with base ABC:

$$V\left(A,B,C\right)=\frac{G\left(d_{A},\;\sigma \right)\tilde{I}\left(A\right)+G\left(d_{B},\;\sigma \right)\tilde{I}\left(B\right)+G\left(d_{C},\;\sigma \right)\tilde{I}\left(C\right)}{3}\cdot S_{ABC}.$$

The first multiplier here is a height of the approximating prism, while the second one is ABC triangle area, computed through coordinates of its corners:

$$S\left(A,B,C\right)=\frac{1}{2}\left|\begin{array}{ccc} A_{x}\; & A_{y}\; & 1\\ B_{x}\; & B_{y}\; & 1\\ C_{x}\; & C_{y}\; & 1 \end{array}\right|.$$

### 3.3. Discrete Differential Operators for Accurate Rasterization

The transition from continuous parametric curves to the discrete pixel grid is a critical step that, if carried out naively, can introduce systematic errors comparable to the inter-fringe spacing in high-density patterns. To achieve sub-pixel accuracy and topological correctness (4-connectivity), we introduce a unified approach based on integer-valued discrete differential operators. These operators analyze the local geometry of the curve directly in pixel-index space, enabling efficient and exact rasterization of both the curve and the surrounding strip.

#### 3.3.1. First-Order Operator: Tangent Direction and Curve Rasterization

Given two consecutive pixel centers, $P_{i-1}=\left(r_{i-1},c_{i-1}\right)$ and $P_{i}=\left(r_{i},c_{i}\right)$, along a discretized curve, we define the first-order discrete directional operator:

$$D_{1}\left(P_{i-1},P_{i}\right)=4+3{\left(\Delta r\right)}^{3}+{\left(\Delta c\right)}^{3},\;\mathrm{where}\;\Delta r=r_{i}-r_{i-1},\;\Delta c=c_{i}-c_{i-1}.$$

**Figure 3:**
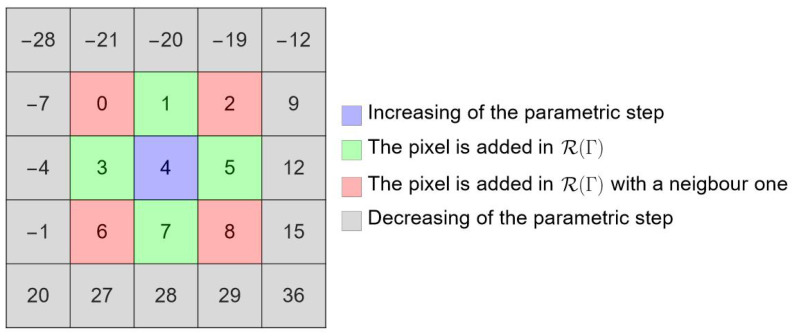
Schematic of the curve rasterization process using a first-order operator $D_{1}$.

This compact formula serves three purposes simultaneously:

- Direction defining: It maps the 9 possible neighbor vectors $\left(\Delta r,\Delta c\right)\in {\left\{-1,0,1\right\}}^{2}$ to unique integers in the range [0,8]. This mapping is shown in Figure 3.
- Neighborhood filter: The sum of the cubic terms, $3{\left(\Delta r\right)}^{3}+{\left(\Delta c\right)}^{3}$ acts as a filter because for $\left(\Delta r,\Delta c\right)\in {\left\{-1,0,1\right\}}^{2}$, the identity $3{\left(\Delta r\right)}^{3}+{\left(\Delta c\right)}^{3}=3\Delta r+\Delta c$ holds, while if a parameter step is too large and causes a jump to a non-adjacent pixel, the sum makes $3{\left(\Delta r\right)}^{3}+{\left(\Delta c\right)}^{3}\geq 8$ (within 2 pixels from $P_{i}$), flagging an invalid “skip”.
- Adaptive step control: This flag triggers an adaptive adjustment of the parametric step Δs: $\Delta s\leftarrow \Delta s/k_{\mathrm{down}}$ if a skip is detected; $\Delta s\leftarrow \Delta s\times k_{\mathrm{up}}$ if $D_{1}=4$ (meaning the parameter advanced but the pixel did not change). Crucially, we use asymmetric coefficients ($k_{\mathrm{up}}=1.5$, $k_{\mathrm{down}}=1.4$) to prevent resonant cycling near stationary points, ensuring robust convergence.

The operator $D_{1}$ is the core of the curve rasterization algorithm. Its scheme is shown in Appendix A (Algorithm A1). The algorithm marches along the continuous curve c(s), adaptively adjusting Δs to produce an ordered, 4-connected list of pixels R. A key subtlety is the handling of diagonal moves: when the curve passes through a pixel corner, there are two equally valid 4-connected paths. In such cases, the algorithm use a special variable, which memorizes the choice made at the previous step, ensuring a smooth, consistent rasterization without jagged artifacts.

#### 3.3.2. Second-Order Operator: Local Curvature and Strip Rasterization

Rasterizing a strip of width 2δ around a curve requires understanding the local curvature to avoid gaps or overlaps in the coverage. Given three consecutive pixel centers $P_{i-1},P_{i},P_{i+1}$ of the already rasterized curve, we define a second-order operator that classifies the local shape. Let $\Delta p_{1}=P_{i}-P_{i-1}$ and $\Delta p_{2}=P_{i+1}-P_{i}$. We define the signs of their components as integers: $s_{x}=\mathrm{sgn}\left(\Delta c_{1}\right)+2\cdot \mathrm{sgn}\left(\Delta c_{2}\right)$ and $s_{y}=\mathrm{sgn}\left(\Delta r_{1}\right)+2\cdot \mathrm{sgn}\left(\Delta r_{2}\right)$. The configuration is then defined as follows:

$$D_{2}=4s_{y}+s_{x}\in \left\{-15,-14,\ldots ,14,15\right\}.$$

After shifting and clamping to the valid range of physically realizable 4-connected triples, we obtain the final formula used in the implementation:

(7) $$D_{2}\left(P_{i-1},P_{i},P_{i+1}\right)=\left\lfloor \frac{15-c_{i-1}-3c_{i}+4c_{i+1}-3r_{i-1}-9r_{i}+12r_{i+1}}{2}\right\rfloor \;\in \left[0,15\right].$$

**Figure 4:**
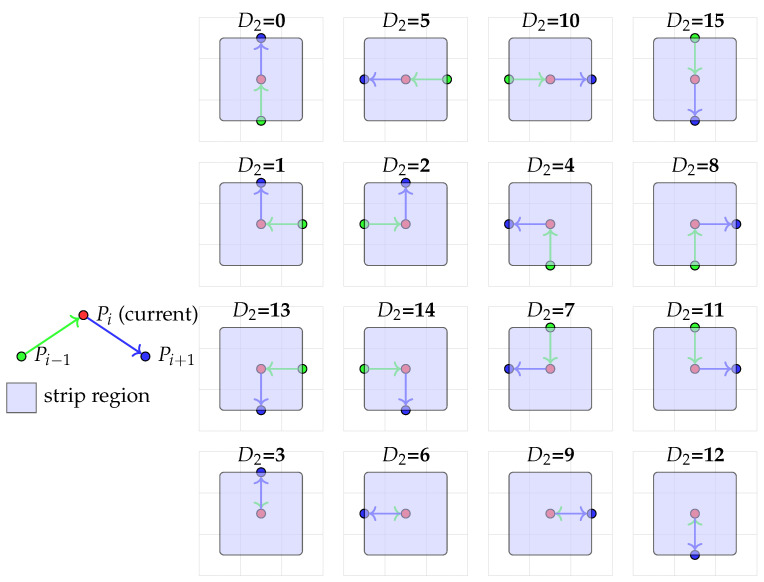
The 16 discrete configurations classified by the second-order operator $D_{2}$ (Equation (7)). For each configuration, the three consecutive curve pixels $P_{i-1}$ (green), $P_{i}$ (red), and $P_{i+1}$ (blue) are shown with arrows indicating the local direction. The shaded blue area illustrates the corresponding region of the strip that needs to be filled. Configurations are grouped: linear motion ($D_{2}\in \left\{0,5,10,15\right\}$), smooth turns ($D_{2}\in \left\{1,2,4,7,8,11,13,14\right\}$), and return points ($D_{2}\in \left\{3,6,9,12\right\}$).

The operator $D_{2}$ evaluates 1 of 16 integer values, each corresponding to a distinct local configuration of the three points (see Figure 4). Geometrically, it approximates the discrete curvature. For example:

- $D_{2}\in \left\{0,5,10,15\right\}$ indicates nearly linear motion (up, left, right, down), warranting a simple rectangular strip fill.
- $D_{2}\in \left\{1,2,4,7,8,11,13,14\right\}$ indicates a smooth turn, requiring a circular sector fill to cover the curved corner of the strip without gaps.
- $D_{2}\in \left\{3,6,9,12\right\}$ corresponds to inflection or “return” points, necessitating a combined fill (e.g., a half-circle plus strips).

Based on the value of $D_{2}$, the strip rasterization algorithm (Algorithm A2 in Appendix A) selects an optimal geometric primitive (line strip or circular arc) and fills the corresponding pixels within distance δ from the central pixel $P_{i}$. A distance mask ensures that if a pixel is covered by multiple primitives, the smallest distance to the central curve is retained, and the corresponding Gaussian weight $\exp(-d^{2}/\left(2\sigma^{2}\right))$ is assigned. This approach guarantees a skip-free, accurate, and weight-aware rasterization of the continuous strip S(γ(θ),δ).

### 3.4. Parametric Models for Fringe Contours

The choice of the parametric subspace $A_{\theta }$ is dictated by the expected physics of the deformation. For a broad class of problems involving axisymmetric or nearly axisymmetric bending of plates and membranes, the fringe contours are expected to be closed, nested, quasi-concentric curves [25]. We introduce two related parametric families that efficiently described such shapes while offering sufficient flexibility to capture deviations from ideal geometry.

#### 3.4.1. Perturbed Circle Model

The most natural model for quasi-circular fringes is a *perturbed circle*, where the constant radius of a circle is replaced by a periodic function of the polar angle φ. In Cartesian coordinates with an origin at $\left(p_{x},p_{y}\right)$, this is expressed as follows:

(8) $$\begin{array}{cc} x(\varphi ) & =p_{x}+R\left(\varphi \right)\cos\varphi ,\\ y(\varphi ) & =p_{y}+R\left(\varphi \right)\sin\varphi , \end{array}$$

where the radius function R(φ) is given by a trigonometric polynomial:

$$R\left(\varphi \right)=r\left[1+\sum_{k=1}^{n}\left(a_{k}\cos\left(\left(k+1\right)\varphi \right)+b_{k}\sin\left(\left(k+1\right)\varphi \right)\right)\right].$$

Here, r>0 is a base radius, $\left(p_{x},p_{y}\right)$ is the center, and ${\left\{a_{k},b_{k}\right\}}_{k=1}^{n}$ are the perturbation coefficients. Setting all $a_{k}=b_{k}=0$ recovers a perfect circle of radius *r* centered at $\left(p_{x},p_{y}\right)$. The shift in harmonic indices (using (k+1)φ instead of kφ) ensures that the lowest-order perturbation already affects the shape, leaving the coefficients $a_{0}$ and $b_{0}$ implicitly absorbed into *r* and the center coordinates.

An equivalent, sometimes computationally convenient, representation expands (8) directly into a Fourier series:

$$\begin{array}{cc} x(\varphi ) & =A_{0}+\sum_{k=1}^{n+1}\left(A_{k}\cos\left(k\varphi \right)+B_{k}\sin\left(k\varphi \right)\right),\\ y(\varphi ) & =C_{0}+\sum_{k=1}^{n+1}\left(C_{k}\cos\left(k\varphi \right)+D_{k}\sin\left(k\varphi \right)\right), \end{array}$$

where the coefficients $\left\{A_{k},B_{k},C_{k},D_{k}\right\}$ are linear combinations of $\left\{r,p_{x},p_{y},a_{k},b_{k}\right\}$. It is simple to show that $A_{0}$, $C_{0}$, and the first harmonics are primarily governed by the “coarse” parameters $r,p_{x},p_{y}$, while higher harmonics depend on the perturbation coefficients. This structure naturally suggests a two-stage optimization strategy:

1. **Quasi-optimization:** vary only the coarse parameters $\left(r,p_{x},p_{y}\right)$ to quickly locate the approximate position and scale of the fringe.
2. **Full optimization:** refine all parameters $\left(r,p_{x},p_{y},\left\{a_{k},b_{k}\right\}\right)$ to capture fine shape details.

The total number of parameters is m=3+2n. In our experiments with plate bending, n=15 (giving 33 parameters) proved sufficient to accurately represent fringes even under significant nonlinear deformation.

#### 3.4.2. Perturbed Ellipse Model

For specimens with pronounced anisotropy or non-axisymmetric boundary conditions, the fringe contours may exhibit a preferred elliptical orientation. The model can be generalized to a *perturbed ellipse* by introducing semi-axes *a* and *b* (a≥b>0) and an orientation angle ψ:

$$\begin{array}{cc} x(\varphi ) & =p_{x}+\rho \left(\varphi ;a,b,\psi \right)\;\left[1+\sum_{k=1}^{n}\left(a_{k}\cos\left(\left(k+1\right)\left(\varphi -\psi \right)\right)+b_{k}\sin\left(\left(k+1\right)\left(\varphi -\psi \right)\right)\right)\right]\cos\varphi ,\\ y(\varphi ) & =p_{y}+\rho \left(\varphi ;a,b,\psi \right)\;\left[1+\sum_{k=1}^{n}\left(a_{k}\cos\left(\left(k+1\right)\left(\varphi -\psi \right)\right)+b_{k}\sin\left(\left(k+1\right)\left(\varphi -\psi \right)\right)\right)\right]\sin\varphi , \end{array}$$

where

$$\rho \left(\varphi ;a,b,\psi \right)=\frac{ab}{\sqrt{\left(b^{2}-a^{2}\right)\cos^{2}\left(\varphi -\psi \right)+a^{2}}}.$$

When a=b=r, this relation reduces to ρ=r, and the model reverts to the perturbed circle (8). The perturbation is now applied relative to the elliptical base shape and is oriented with it (via the phase shift φ−ψ). The coarse parameter set for this model is $\left(a,b,\psi ,p_{x},p_{y}\right)$, totaling 5+2n parameters.

#### 3.4.3. Parameter Constraints and Initialization

To ensure physical plausibility and improve optimization convergence, constraints are applied: r,a,b>0; perturbation coefficients are bounded ($|a_{k}\left|,\right|b_{k}|<0.5$ to prevent self-intersection); and for the ellipse, a≥b. The initial guess for the first (innermost) fringe is obtained via a coarse search: the image center is estimated from intensity function cross-sections (φ=const); then, *r* (and a,b later) is varied until a local extremum of $J^{s}$ is found. For subsequent fringes, the parameters of the previous fringe are scaled outward by a factor slightly greater than one to provide a good starting point for the quasi-optimization step.

The fringe limiting density is governed by image resolution and the strip integration width. For stable algorithm operation, the minimum distance between adjacent fringes should exceed twice the integration half-width (2δ). With our standard choice of δ=3 pixels (which ensures averaging over several speckles), this requires an inter-fringe distance of at least 6 pixels. In practice, with moderate noise, the algorithm works successfully even at distances of about δ = 5–15 pixels.

These parametric models, combined with the strip integration functional and the rasterization operators, constitute the proposed skeletonization framework. However, before proceeding to the experimental validation of its performance in Section 4, it is necessary to prepare the intensity matrix to reduce the influence of speckle noise.

### 3.5. Local Intensity Equalization Algorithm

Raw interferograms often exhibit slow spatial variations in background intensity and contrast due to uneven illumination and polarization effects (see Section 2.1). To normalize these variations without distorting the fringe positions, we employ a local intensity equalization procedure based on quantile statistics within sliding windows.

#### 3.5.1. Local Quantile Computation

**Figure 5:**
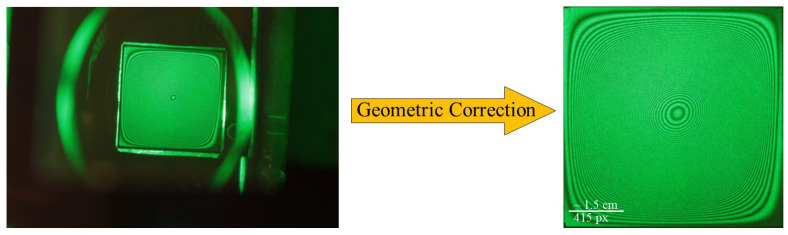
Original image with a projected grid superimposed for distortion assessment and the image after geometric correction via projective transformation.

Before processing the intensity matrix, it is necessary to remove areas of the image without fringes, retaining only the useful region. Furthermore geometric distortion correction must be applied to compensate for the misalignment between the camera lens axis and the object beam. An example of this procedure, which we will call the geometric correction, is shown in Figure 5.

Let I[r,c] denote the geometrically corrected intensity matrix of size $r_{\max}\times c_{\max}$. For each pixel $\left(r_{q},c_{q}\right)$ considered a potential window center, we define a local block $M_{r_{q},c_{q},q}$ of size q×q:

$$M_{r_{q},c_{q},q}\left[i,j\right]=I\left[R\left(r_{q}+i-\left\lfloor q/2\right\rfloor \right),\;C\left(c_{q}+j-\left\lfloor q/2\right\rfloor \right)\right],\;i,j=1,\ldots ,q,$$

where R(k) and C(k) are reflection-padding functions that handle boundary pixels:

$$R\left(k\right)=\left\{\begin{array}{cc} k, & 1\leq k\leq r_{\max}\\ 1-k, & k<1\\ 2r_{\max}-k, & k>r_{\max} \end{array}\right.,\;C\left(k\right)=\left\{\begin{array}{cc} k, & 1\leq k\leq c_{\max}\\ 1-k, & k<1\\ 2c_{\max}-k, & k>c_{\max} \end{array}\right..$$

This padding ensures that windows near image boundaries remain fully populated without introducing artificial discontinuities.

For each block $M_{r_{q},c_{q},q}$, we compute two statistics: the lower quantile $a[r_{q},c_{q}]$ and the upper quantile $b[r_{q},c_{q}]$ at probability levels ν and 1−ν, respectively (typically ν=0.05). These quantiles estimate the local minimum and maximum intensity while being resistant to outlier pixels caused by speckle noise:

$$a\left[r_{q},c_{q}\right]=Q_{\nu }\;\left(M_{r_{q},c_{q},q}\right),\;b\left[r_{q},c_{q}\right]=Q_{1-\nu }\;\left(M_{r_{q},c_{q},q}\right).$$

#### 3.5.2. Interpolation and Normalization

The quantiles a[r,c] and b[r,c] are computed only on a coarse grid with spacing d=⌊q/2⌋ to reduce computational cost. To obtain values at every pixel (i,j), we use linear interpolation:

$$\hat{a}\left(i,j\right)=\mathrm{Int}\left(\begin{array}{cccc} a\left[1,1\right]\; & a\left[1,d+1\right]\; & \ldots \; & a\left[1,c_{\max}\right]\\ a\left[d+1,1\right]\; & a\left[d+1,d+1\right]\; & \ldots \; & a\left[d+1,c_{\max}\right]\\ \ldots \ldots & \ldots \ldots & \ldots \ldots & \ldots \ldots \\ a\left[r_{\max},1\right]\; & a\left[r_{\max},d+1\right]\; & \ldots \; & a\left[r_{\max},c_{\max}\right] \end{array}\right),$$

$$\hat{b}\left(i,j\right)=\mathrm{Int}\left(\begin{array}{cccc} b\left[1,1\right]\; & b\left[1,d+1\right]\; & \ldots \; & b\left[1,c_{\max}\right]\\ b\left[d+1,1\right]\; & b\left[d+1,d+1\right]\; & \ldots \; & b\left[d+1,c_{\max}\right]\\ \ldots \ldots & \ldots \ldots & \ldots \ldots & \ldots \ldots \\ b\left[r_{\max},1\right]\; & b\left[r_{\max},d+1\right]\; & \ldots \; & b\left[r_{\max},c_{\max}\right] \end{array}\right).$$

The normalized intensity at each pixel is then obtained by linear rescaling:

$$\tilde{I}\left[i,j\right]=\frac{I\left[i,j\right]-\hat{a}\left(i,j\right)}{\hat{b}\left(i,j\right)-\hat{a}\left(i,j\right)}.$$

This operation maps the local intensity range $\left[\hat{a}\left(i,j\right),\hat{b}\left(i,j\right)\right]$ approximately to [0,1], effectively compensating for low-frequency inhomogeneities while preserving the high-frequency fringe structure.

#### 3.5.3. Parameter Selection

The window size *q* is chosen to be several times larger than the expected inter-fringe distance but smaller than characteristic scales of illumination non-uniformity. In our experiments with square plates, q=31 pixels (covering about 3–4 fringes) worked well. The quantile level ν=0.05 provides a good compromise between rejecting speckle outliers and retaining true fringe extrema. The equalization is applied once after geometric correction and, if needed, repeated after optional Fourier filtering to compensate for global intensity shifts introduced by the filter.

### 3.6. Complete Skeletonization Algorithm

**Figure 6:**
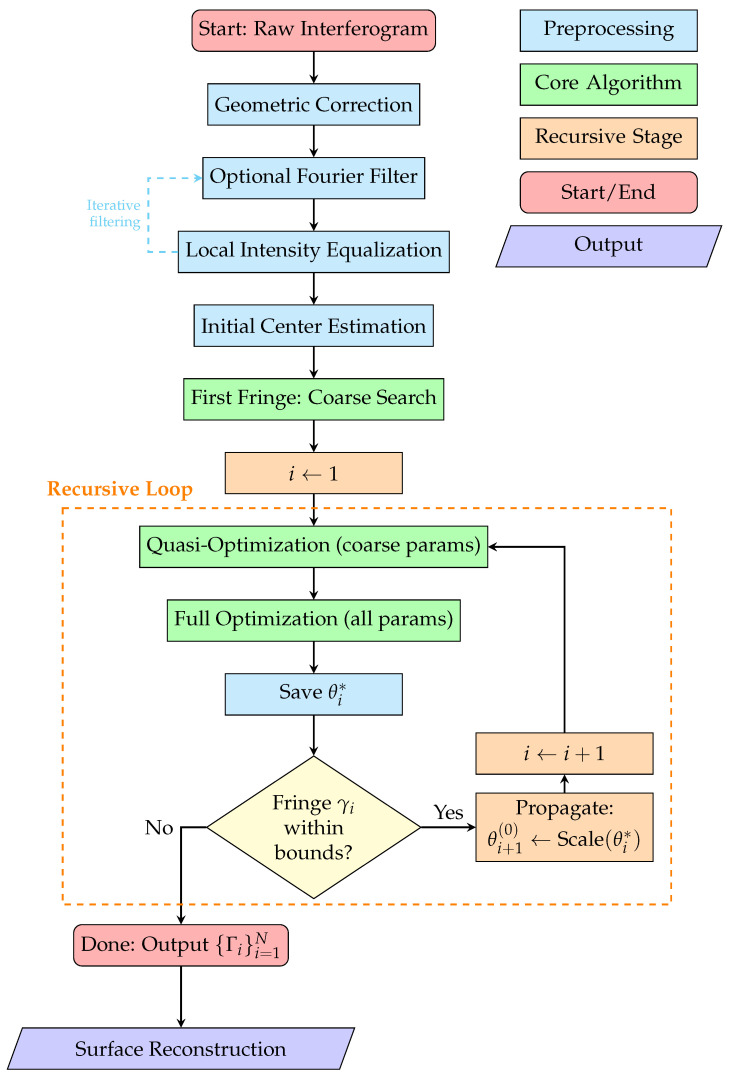
Flowchart of the complete skeletonization algorithm. Blocks are color-coded according to their functional role: blue for pre-processing steps, green for core algorithmic stages, orange for the recursive optimization loop, red for start/end points, and cyan for output/reconstruction. The color key is provided in the upper-right corner. The dashed arrow indicates an optional iterative cycle of filtering and equalization for images with extreme noise levels.

Integrating all components described in the previous subsections, we present the complete procedure for automated fringe skeletonization. The algorithm proceeds recursively from the innermost to the outermost fringe, taking into account the quasi-similarity property. The main steps are illustrated in Figure 6. The algorithm iteratively identifies fringes until one of the three termination conditions is met:

- The last computed intensity integral is equal to zero;
- The last identified fringe lies outside the image boundaries;
- The required (or preset) number of fringes has been identified.

### 3.7. Synthetic Interferogram Generation

To quantitatively validate the proposed algorithm under controlled conditions, we developed a virtual interferogram generator that produces images with known pattern geometry and physically realistic noise characteristics. The generator takes into account two key aspects: (1) the typical fringe pattern of a bent square plate, and (2) the corruption mechanisms described in Section 2.1.

#### 3.7.1. Ideal Fringe Geometry via Morphing

The underlying displacement field is modeled by a family of closed curves that morph smoothly from a circle at the center to a square at the boundary, parameterized by a morphing parameter α∈[0,1]. In the first octant (φ∈[0,π/4]), the curve consists of a straight segment and a curved segment that ensures $C^{1}$ continuity:

$$\begin{array}{cccc} r_{s}\left(\varphi ;\alpha \right) & =\alpha \sec\varphi , & \varphi & \in \left(0,\;\alpha \pi /4\right],\\ r_{c}\left(\varphi ;\alpha \right) & =\alpha \left[\frac{2}{\pi (1-\alpha )}\left(\frac{\alpha \pi }{4}-\varphi \right)\left(\frac{\alpha \pi }{4}+\varphi -\frac{\pi }{2}\right)\tan\frac{\alpha \pi }{4}+1\right]\sec\frac{\alpha \pi }{4}, & \varphi & \in \left(\alpha \pi /4,\;\pi /4\right]. \end{array}$$

The full curve over all angles is obtained by symmetric replication. Equivalently, it can be expressed compactly as follows:

$$r\left(\varphi ;\alpha \right)=\left\{\begin{array}{cc} \alpha \;r_{1}\;\left(\varphi \;\mathrm{mod}\;\frac{\pi }{2},\;\frac{\pi \alpha }{4}\right), & 0<\varphi \;\mathrm{mod}\;\frac{\pi }{2}\leq \frac{\pi }{4},\\ \alpha \;r_{2}\;\left(\varphi \;\mathrm{mod}\;\frac{\pi }{2},\;\frac{\pi }{4}\left(2-\alpha \right)\right), & \frac{\pi }{4}<\varphi \;\mathrm{mod}\;\frac{\pi }{2}\leq \frac{\pi }{2}, \end{array}\right.$$

where

$$\begin{array}{cc} \; & r_{1}\left(\varphi ,\alpha \right)=\left\{\begin{array}{cc} \left[\frac{\alpha -\varphi }{\pi -4\alpha }\left(2\alpha +2\varphi -\pi \right)\tan\alpha +1\right]\sec\alpha ,\; & \varphi >\alpha \\ \sec\varphi \; & \varphi \leq \alpha \end{array}\right.,\\ \; & r_{2}\left(\varphi ,\alpha \right)=\left\{\begin{array}{cc} \csc\varphi ,\; & \varphi >\alpha \\ \left[\frac{\alpha -\varphi }{\pi -4\alpha }\left(\pi -2\alpha -2\varphi \right)\cot\alpha +1\right]\csc\alpha ,\; & \varphi \leq \alpha \end{array}\right.. \end{array}$$

The ideal, noise-free intensity pattern is then defined as a periodic function of α:

$$I_{\mathrm{ideal}}\left(x,y\right)=\sin^{2}\left(k\pi \alpha (x,y)\right),$$

where *k* controls the fringe density. The mapping (x,y)↦α is obtained by numerically solving the transcendental equation:

$$r\left(\varphi ,\;\alpha \right)=\sqrt{x^{2}+y^{2}},$$

for α at each grid point.

#### 3.7.2. Physically Motivated Noise Model

The ideal intensity is corrupted by two noise sources derived from the physical model in Section 2.1, which presents the general formula for corrupted intensity (1); however, the terms describing speckle noise were not specified. Accurately defining these terms is a complicated problem, which has considered in a numerous studiess (e.g., [23,26,27]). For this paper, it is enough to note that speckle noise consists of three parts, which, related to reflected surface factor, depend on its roughness, spatial and temporal coherence:

$$N_{\mathrm{temp}}=1+{\left(2\Delta L\Delta \nu /c\right)}^{2},\;N_{\mathrm{spat}}=1+{\left(D/\rho_{\mathrm{speckle}}\right)}^{2},$$

where *c* is the speed of light in vacuum, Δν is the laser spectral linewidth, ΔL the path length difference, *D* is the diameter of the illuminated object area, and $\rho_{\mathrm{speckle}}$ is the characteristic speckle size:

$$\rho_{\mathrm{speckle}}\approx \max\left(\lambda z/D,\;L_{c}z/D\right).$$

Here, λ is the laser wavelength, *z* is the distance from object to observation plane and $L_{c}$ is the reflected surface roughness parameter. The resulting speckle noise can be modeled as the product of different coherence terms and the surface factor, which is obtained empirically.

In practice, for rapid commutation and parameter studies, it is convenient to employ a simplified combined noise model, constructed as stochastic perturbations of phase difference and amplitude:

(9) $$\tilde{I}\left(x,y\right)=I_{0}\left(x,y\right)+k\nu \left(x,y\right)+2\sqrt{k\nu \left(x,y\right)I_{0}\left(x,y\right)}\cos\left(\delta \pi \right),$$

where *k* controls the overall noise level, and ν(x,y), δ(x,y) are independent stochastic processes with correlation:

$$\langle f\left(x\right)f\left(x^{\prime }\right)\rangle ={\left[2J_{1}\left(\pi |x-x^{\prime }|/\rho_{\mathrm{speckle}}\right)/\left(\pi |x-x^{\prime }|/\rho_{\mathrm{speckle}}\right)\right]}^{2},$$

where $J_{1}\left(\cdot \right)$ is the Bessel function. This model approximates the statistical properties of speckle while remaining computationally efficient.

#### 3.7.3. Calibration of Noise Models

For systematic performance evaluation, we employ a simplified linear mixing model derived from the physical model (9). Under the normalization conditions $\langle I_{0}\rangle =1$ (ideal signal normalized to unit mean) and 〈ν〉=0, $\langle \nu^{2}\rangle =1$ (noise with zero mean and unit variance), the two models are related by an exact linear transformation. Defining the normalized observed intensity $\hat{I}=\tilde{I}/\left(1+k\right)$, we obtain the linear mixing model:

(10) $$\hat{I}=\left(1-\alpha \right)I_{0}+\alpha \nu_{\mathrm{norm}},$$

where α=k/(1+k) and $\nu_{\mathrm{norm}}=\nu /\sqrt{\langle \nu^{2}\rangle }$. Here, α∈[0,1] represents the noise fraction, with α=0 corresponding to the ideal pattern and α=1 to pure noise. This exact correspondence ensures that both models yield identical first- and second-order intensity statistics under the stated normalization.

Throughout this work, references to specific noise levels (e.g., “95% noise”) correspond to α=0.95 in (10), equivalent to k=19 in (9). The linear model provides intuitive control over the signal-to-noise ratio, where SNR≈(1−α)/α.

#### 3.7.4. Spatial Correlation of Speckle Noise

Real speckle patterns exhibit spatial correlation over a characteristic length scale $\rho_{\mathrm{speckle}}$ (speckle size). To replicate this property, our synthetic noise field ν(x,y) is generated via a two-stage process:

1. An initial uncorrelated random field $\nu_{0}\left(x,y\right)$ is sampled from an appropriate distribution.
2. This field is convolved with a Gaussian kernel $G_{\sigma_{c}}$ of width $\sigma_{c}\propto \rho_{\mathrm{speckle}}$:
  $$\nu \left(x,y\right)=\left(\nu_{0}*G_{\sigma_{c}}\right)\left(x,y\right).$$

The resulting field has the desired spatial correlation, approximating the typical speckle autocorrelation function. The strip integration functional is particularly effective against such correlated noise because its integration width δ is chosen to exceed $\rho_{\mathrm{speckle}}$, enabling averaging over multiple correlation cells and thus significant noise suppression while preserving the fringe signal.

The characteristic speckle size $\rho_{\mathrm{speckle}}$ used in our synthetic noise model is informed by experimental measurements of real speckle patterns under various conditions. While real speckle exhibits multi-scale structure, our single-scale correlated noise captures the essential spatial correlation that affects fringe detection algorithms. The Gaussian convolution approximates the theoretically expected Bessel-type correlation, with the kernel width calibrated to match the measured correlation length.

#### 3.7.5. Error Metrics

For each synthetic image, the exact curves $\left\{\gamma_{i}^{\mathrm{true}}\right\}$ are known analytically. This allows us to define rigorous error metrics for any skeletonization result $\left\{\gamma_{i}^{\mathrm{res}}\right\}$:

$$\begin{array}{cccc} \; & \mathrm{Euclidean}\mathrm{metric}: & \; & \;\varepsilon_{\mathrm{Euc}}=\sqrt{\frac{1}{L^{\mathrm{true}}}\int_{0}^{2\pi }\left[{\left(x^{\mathrm{true}}\left(\varphi \right)-x^{\mathrm{res}}\left(\varphi \right)\right)}^{2}+{\left(y^{\mathrm{true}}\left(\varphi \right)-y^{\mathrm{res}}\left(\varphi \right)\right)}^{2}\right]\;d\varphi },\\ \; & \mathrm{Max}\mathrm{error}: & \; & \;\varepsilon_{\max}=\frac{\max_{\varphi \in \left[0,\;2\pi \right)}\sqrt{{\left(x^{\mathrm{true}}\left(\varphi \right)-x^{\mathrm{res}}\left(\varphi \right)\right)}^{2}+{\left(y^{\mathrm{true}}\left(\varphi \right)-y^{\mathrm{res}}\left(\varphi \right)\right)}^{2}}}{\max_{\varphi \in \left[0,\;2\pi \right)}\sqrt{{\left(x^{\mathrm{true}}\left(\varphi \right)\right)}^{2}+{\left(y^{\mathrm{true}}\left(\varphi \right)\right)}^{2}}}. \end{array}$$

These metrics are used in Section 4 to quantitatively compare the performance of different methods.

### 3.8. Implementation Details

The described skeletonization procedure has been implemented in a custom C++ program. This program is capable of performing all steps of the proposed algorithm (see Figure 6):

1. Geometric correction;
2. Filtering in the frequency domain with different kernels;
3. Intensity equalization, as described in Section 3.5;
4. Localization of the image center;
5. Recursive identification of fringes, which includes the following aspects:
  (a) Initializing the parametric curve (Section 3.4.1 and Section 3.4.2);
  (b) Computing the intensity integral (Section 2.4) using numerical quadratures (Section 3.2);
  (c) Defining the approximation curve’s parameters.

After extensive testing of several optimization approaches, we developed a tailored strategy that balances efficiency and robustness:

**Optimization framework:** the identification of each fringe involves two optimization stages:

1. **Coarse quasi-optimization:** A modified coordinate descent algorithm with adaptive step sizing and momentum (e.g., [28]) accelerates the initial search for the fringe’s approximate position and scale, varying only the geometric parameters (center coordinates and base radius/axes).
2. **Fine refinement:** A conjugate gradient method with dynamic parameter scaling performs the full optimization of all parameters, including the higher-order Fourier perturbation coefficients. Analytic gradients of the strip integration functional $J^{s}$ are computed via automatic differentiation.

**Parameters and stopping criteria:** Practical step sizes were determined empirically: 0.5 pixels for geometric parameters, 0.001 for normalized Fourier coefficients. Optimization terminates when either the relative change in $J^{s}$ falls below $10^{-6}$ or the norm of the parameter update drops below $10^{-4}$.

**Risk control and convergence safeguards:** A primary challenge in real interferograms is the presence of intensity plateaus caused by pre-processing or extreme noise. Our implementation includes the following:

- **Plateau detection:** if the objective function shows negligible improvement (<$10^{-8}$) over 20 consecutive iterations, stagnation is flagged.
- **“Shaking” recovery:** upon stagnation, parameters receive a small random perturbation (5–10% of the current step size), and optimization restarts from this perturbed state.
- **Dynamic variable prioritization:** the algorithm tracks parameter sensitivity and temporarily focuses the search on the most influential variables when progress slows.

In systematic tests, this approach converged to physically plausible fringe contours in over 98% of cases (6000 individual fringe extraction attempts, 100 synthetic 512×512 pixel interferograms × average 60 fringes each). The remaining failures occurred only with grossly incorrect initialization (e.g., center placed outside the fringe pattern), underscoring the importance of the coarse center estimation step described in Section 3.5. The algorithm fails predominantly in two scenarios: (1) at moderate noise (α=0.4) with very dense fringes (80 fringes, spacing 3 pixels), or (2) at high noise (α=0.9) with moderate fringe density (20 fringes). In both cases, the intensity modulation between adjacent fringes becomes insufficient for reliable separation.

**Figure 7:**
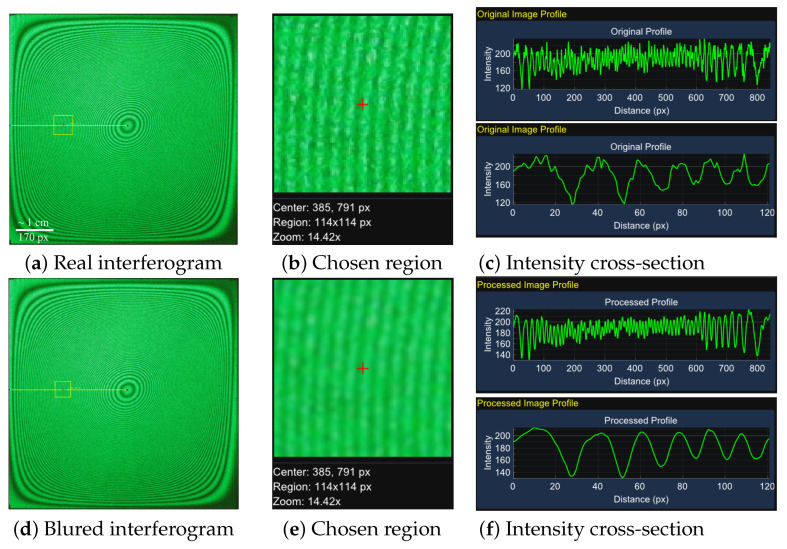

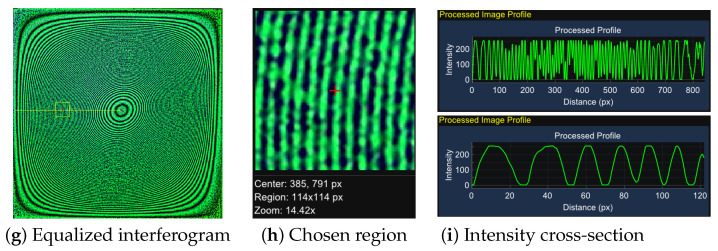
The pre-processing of a real interferogram. Rows (top to bottom): (**a**–**c**) original image; (**d**–**f**) image after Gaussian filtering (blur); (**g**–**i**) image after intensity equalization. Columns (left to right): (**a**,**d**,**g**) full image; (**b**,**e**,**h**) magnified chosen region; (**c**,**f**,**i**) intensity cross-section, showing the original function (top) and the function after removal of false extrema (bottom).

An example of the pre-processing of a real interferogram, performed using the developed program, is shown in Figure 7.

Apart from that, the program contains a set of standard tools for working with images and includes a custom generator of synthetic fringe patterns, a described in Section 3.7. The generator accepts the following control parameters:

- Fringe density *k* (number of fringes across the field);
- Speckle noise, dependent on surface roughness parameter and the laser spectral linewidth;
- Noise models the influence of non-uniform illumination.

For each generated image, the exact curves $\left\{\gamma_{i}^{\mathrm{true}}\left(\alpha =i/k\right)\right\}$ are known analytically. This allows for the quantification of the algorithm errors.

## 4. Validation on Synthetic Interferograms

### 4.1. The Efficiency of Fringe Identification with Different Variants of the Intensity Integral

**Figure 8:**
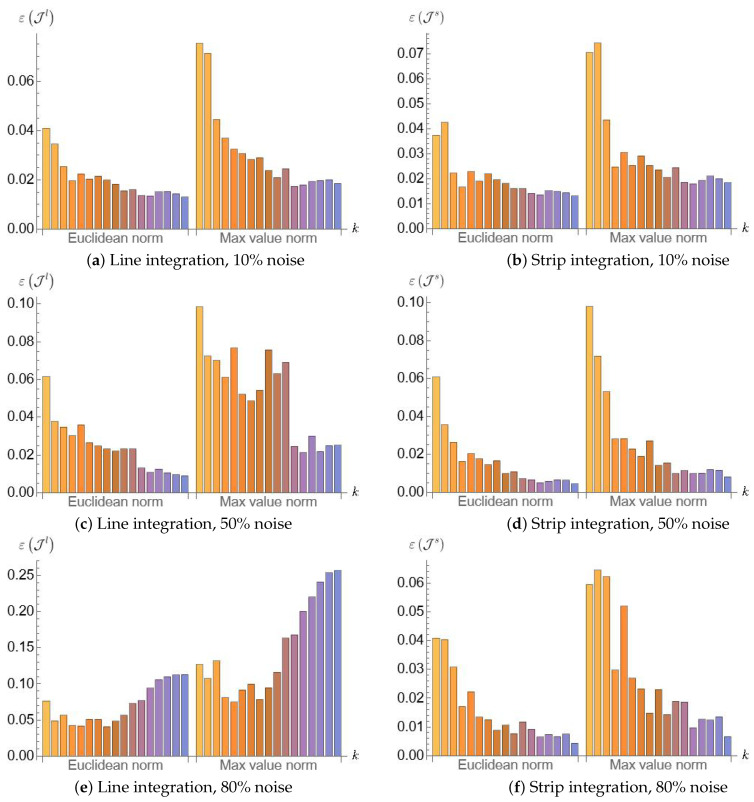
A comparison of identification efficiency for different integration methods.

**Figure 9:**
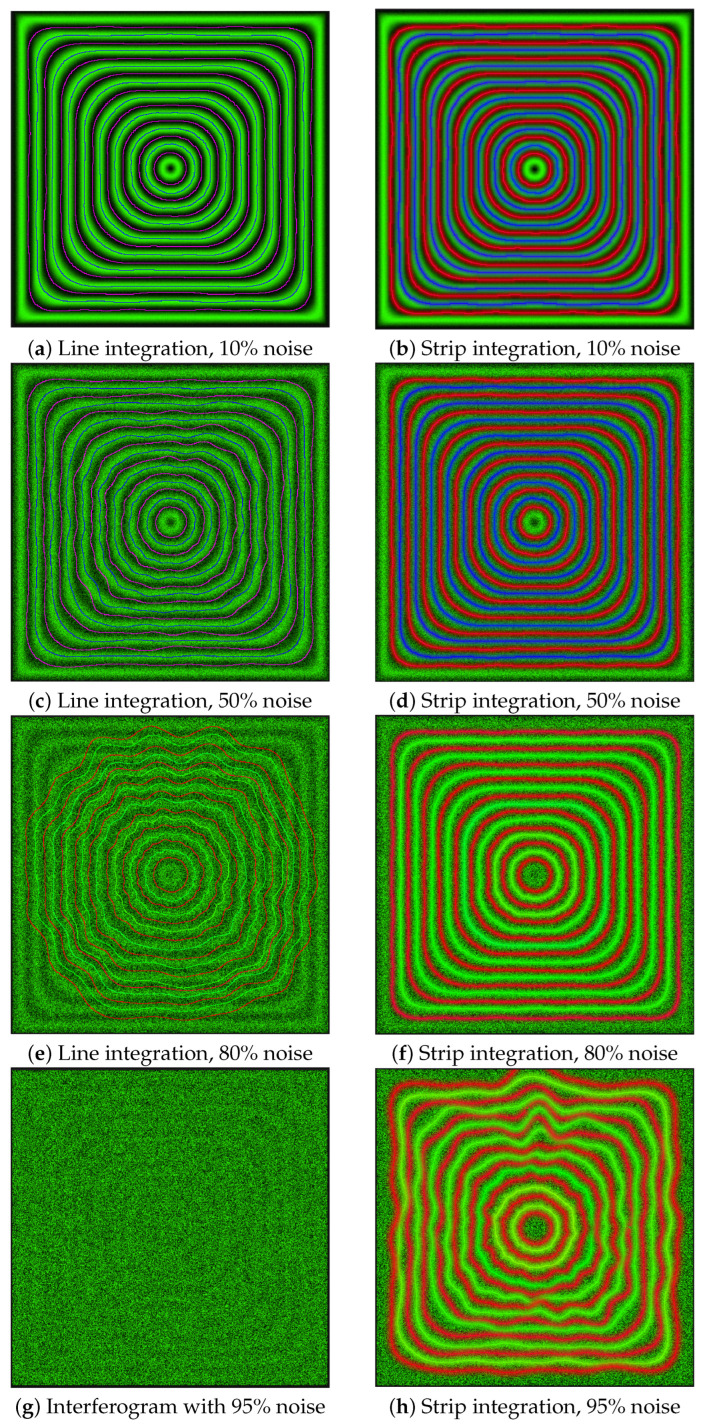
Fringe identification from synthetic interferogram (512×515 px) by line integration and strip integration with different noise levels.

We compared the efficiency of fringe identification with different variants of the intensity integral, using the metrics defined in Section 3.7.5. Figure 8 demonstrates that with slightly noisy images, both methods have similar performance, whereas for highly noisy patterns, the strip integration method has a significant advantage over the line integration one. Moreover, as is shown in Figure 9, the strip integration method is capable of identifying fringes in images with noise levels as high as 95%, where it is hardly possible to distinguish the fringes even visually.

### 4.2. Why General-Purpose Methods Fail: A Fundamental Analysis

A meaningful comparison with baseline methods must first address why standard approaches are fundamentally mismatched to the problem of high-density, high-noise fringe skeletonization. Consider the two most relevant classes, as detailed below.

#### 4.2.1. Edge and Ridge Detection (Canny, Shen–Castan)

**Figure 10:**
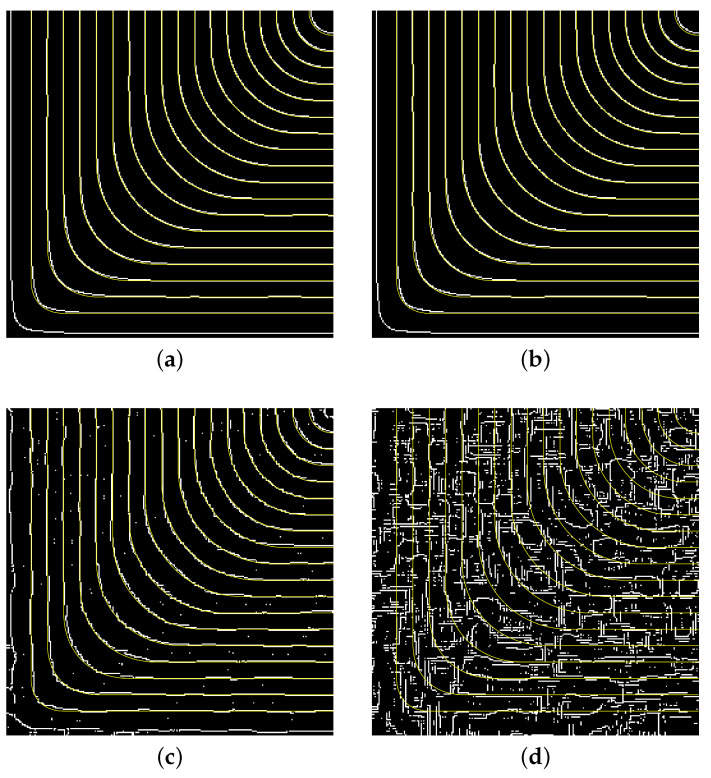
Edge detection results on synthetic interferograms. Yellow curves show exact intensity gradient maxima (theoretical edge positions); white pixels show detected edges. Even at moderate noise, detected edges are fragmented and offset from true fringe centers. (**a**) 10% noise, 95% $R_{3}$ coverage. (**b**) 50% noise, 90% $R_{3}$ coverage. (**c**) 80% noise, 70% $R_{3}$ coverage. Significant fragmentation. (**d**) 95% noise, 10% $R_{3}$ coverage. Total fragmentation.

These methods are designed to detect *discontinuities* or *gradient maxima*. For fringe patterns, they produce discrete sets of edge pixels (Figure 10). Even under moderate noise, the output is fragmented and lacks topological structure. Converting this pixel cloud into a family of smooth, nested, closed curves requires additional heuristic processing that is both complex and unreliable, especially when fringes are closely spaced.

#### 4.2.2. Active Contour Models (Snakes)

Standard snake formulations minimize an energy functional $E=E_{\mathrm{int}}+E_{\mathrm{ext}}$, where $E_{\mathrm{ext}}$ typically attracts the contour to *image gradients* (edges). To adapt a snake to locate fringe *centers* (intensity extrema), one would need to:

1. Redefine $E_{\mathrm{ext}}$ to target intensity extrema rather than gradients.
2. Replace pointwise gradient computation with a *strip integration* functional for noise robustness.
3. Ensure functional smoothness via bicubic interpolation to enable gradient-based optimization.
4. Constrain the snake to a parametric subspace (e.g., Fourier-based curves) to guarantee correct topology.
5. Implement recursive initialization leveraging fringe similarity for efficiency.

These modifications would essentially recreate the core components of our proposed method. The “baseline” snake would cease to be a general-purpose tool and become a specialized implementation of our approach.

#### 4.2.3. Quantitative Assessment of Edge Detection

Edge detection algorithms (such as Canny) are fundamentally designed to locate *gradient extrema*—points of maximum intensity change, which correspond to transitions between bright and dark regions. In an ideal fringe pattern, these would lie midway between adjacent fringe centers. Our method, in contrast, directly targets the *intensity extrema* (fringe centers). This conceptual mismatch means edge detectors cannot directly produce the skeleton needed for displacement field reconstruction, even under ideal conditions.

Despite this fundamental difference, we can define a rough success metric to assess how well edge detection “covers” the true fringe locations: for each ground-truth fringe curve, we compute the percentage of its pixels that lie within a *d*-pixel neighborhood (d=3) of any detected edge pixel. This *fringe coverage rate* $R_{d}$ measures the geometric proximity of detected edges to true fringe centers.

As Figure 10 shows, edge detection performs reasonably under low noise but produces fragmented, offset edge maps. At 80% noise, fragmentation becomes severe, and at 95% noise, the coverage rate drops below 10%. More importantly, even when edges are detected, they represent gradient maxima between fringes, not the intensity extrema required for displacement measurement. This fundamental limitation, combined with noise sensitivity, makes edge detection unsuitable for high-precision skeletonization.

## 5. Application to Real High-Density Interferograms

**Figure 11:**
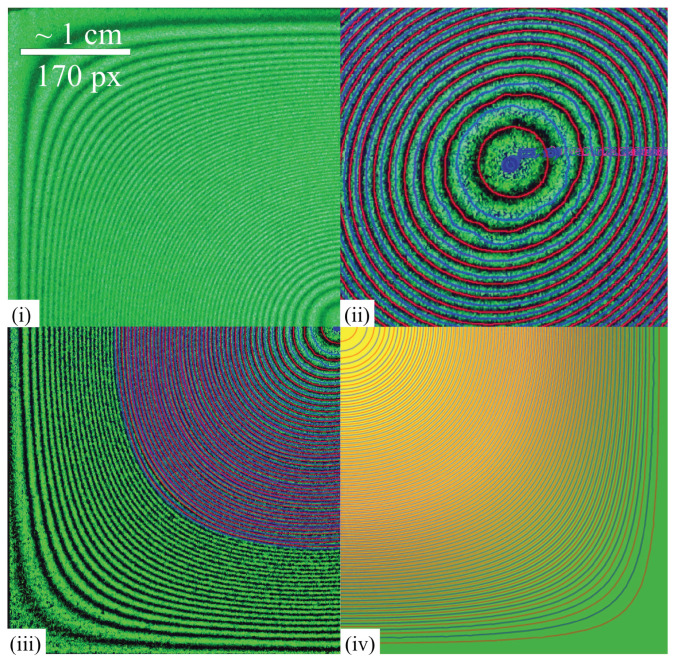
Validation of the proposed procedure on a real hologram. (**i**) Region of the original hologram. (**ii**) Magnified central region of the processed image. (**iii**) Full processed image, displaying both the identified fringes (red and blue lines) and the intensity-equalized fringe pattern. (**iv**) Deformed surface of the plate obtained from independent theoretical modeling for comparison.

To validate the proposed algorithms, these were tested on a real hologram, obtained from a bending experiment on a square plate. The plate (copper, side length 6 cm, thickness 184 µm) was subjected to a uniform transverse load in a holographic interferometry setup (Leith–Upatnieks off-axis scheme with a solid-state laser, λ=532 nm). Interferograms were recorded for several load increments. The most challenging case, with a deflection generating over 100 fringes (shown in Figure 11(i)), was selected for analysis.

The set of identified fringes (red and blue lines in Figure 11(ii,iii) was compared with the results (Figure 11(iv)), obtained from theoretical modeling, presented in [22]. As shown in Figure 11, the identified fringes are in good agreement with isolines of deformed surface, demonstrating the acceptable accuracy of the proposed procedure. The RMS deviation between the displacement field reconstructed from our new algorithm’s skeleton and the independent theoretical model is 0.12λ (≈64 nm).

## 6. Conclusions

This paper presents a novel, robust method for skeletonizing fringe patterns in holographic interferometry. By constraining the solution to a physics-informed parametric subspace and employing a strip integration functional, the method achieves high accuracy and stability in the presence of strong speckle noise, where conventional techniques may fail. Quantitative validation on synthetic data demonstrated a significant reduction in error compared to baseline methods (Canny edge detection and GVF snakes). Practical utility was confirmed by successfully processing a real interferogram with over 100 fringes, the results of which closely matched those of an independent theoretical model.

### Applicability, Limitations, and Future Work

The presented algorithm is specifically tailored for analyzing interferograms where the fringe family exhibits a physically motivated, simple topology – specifically, families of closed, nested, and quasi-similar contours. This pattern is characteristic of axisymmetric or quasi-axisymmetric bending in plates and membranes with convex boundaries (circular, square, polygonal), which are common test objects in the experimental mechanics of MEMS elements and thin-film structures [29].

The main limitations of the current implementation stem directly from its core design choices:

- Topological constraints: The algorithm is most effective when the fringe topology is known a priori to consist of nested, simply connected curves. It may encounter difficulties or produce suboptimal results for patterns featuring fringe bifurcations (e.g., wrinkling patterns [30]), interruptions (e.g., near cracks or holes), or multiple disconnected families without a single dominant center.
- Initialization dependency: The recursive propagation strategy requires a reasonable initial guess for the innermost fringe (coarse center estimation). While the algorithm includes a robust coarse search, a severely erroneous initialization (e.g., outside the fringe field) may prevent convergence.
- Quasi-similarity assumption: The recursive search relies on the geometric similarity of adjacent fringes. A drastic, abrupt change in fringe shape between consecutive contours violates this assumption and could cause the propagation to fail or jump to an incorrect fringe.

Pathways for generalization and future work: the limitations outlined above define clear directions for extending the framework:

1. Handling complex topologies: For patterns with bifurcations or multiple centers, a pre-processing segmentation step could partition the image into regions, each containing a fringe family with simple topology. The proposed algorithm could then be applied independently within each region.
2. Robustness enhancement via functional modification: The risk of the algorithm “jumping” to an adjacent fringe or failing on complex patterns could be mitigated by augmenting the strip integration functional $J^{s}$ with additional regularization terms. For instance, terms that penalize excessive variation in intensity along the strip centerline could help detect anomalies and prevent incorrect convergence.
3. Automated model selection: Future developments could include a preliminary analysis stage to automatically infer the appropriate parametric model (e.g., circle vs. ellipse, required Fourier order) and topology from the raw interferogram, reducing the need for manual parameter selection.

Despite these limitations, the proposed method provides a powerful and reliable tool for a specific but critically important class of problems in optical metrology. Its ability to deliver sub-pixel accuracy from single, extremely noisy interferograms makes it a valuable asset for experimentalists studying high-sensitivity deformation phenomena.

The general principles of the method—parametric modeling of expected topology combined with robust integration for noise suppression—could potentially inspire adaptations for other pattern analysis problems, such as processing certain types of wrinkle or surface topography images. However, such applications would require careful consideration of domain-specific noise characteristics and topological constraints, which lie outside the scope of this holography-focused work.

## Data Availability

Data are contained within this article.

## Appendix A. Rasterization Algorithms Pseudocode

**Algorithm A1:** Adaptive Curve Rasterization using First-Order Operator $D_{1}$

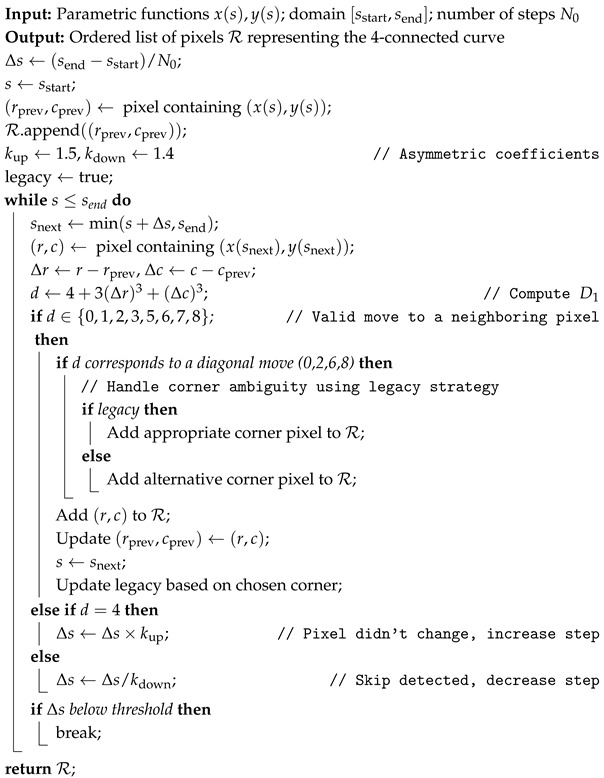

**Algorithm A2:** Strip Rasterization using Second-Order Operator $D_{2}$

## References

[1] Kobayashi A. (1987). Handbook on Experimental Mechanics.

[2] Vest C. (1979). Holographic Interferometry.

[3] Malacara D., Servin M., Malacara Z. (2005). Interferogram Analysis for Optical Testing.

[4] Kreis T. (2006). Handbook of Holographic Interferometry: Optical and Digital Methods.

[5] Distante A., Distante C. (2020). Handbook of Image Processing and Computer Vision: Volume 1: From Energy to Image.

[6] Schwider J., Falkenstoerfer O.R., Schreiber H., Zoeller A., Streibl N. (1993). New compensating four-phase algorithm for phase-shift interferometry. Opt. Eng..

[7] Zuo C., Feng S., Huang L., Tao T., Yin W., Chen Q. (2018). Phase shifting algorithms for fringe projection profilometry: A review. Opt. Lasers Eng..

[8] Tabata S., Maruyama M., Watanabe Y., Ishikawa M. (2019). Pixelwise Phase Unwrapping Based on Ordered Periods Phase Shift. Sensors.

[9] Marr D., Hildreth E. (1980). Theory of edge detection. Proc. R. Soc. Lond. Ser. B Biol. Sci..

[10] Canny J. (2009). A computational approach to edge detection. IEEE Trans. Pattern Anal. Mach. Intell..

[11] Shen J., Castan S. (1992). An optimal linear operator for step edge detection. CVGIP Graph. Model. Image Process..

[12] Xu C., Prince J.L. (1997). Gradient vector flow: A new external force for snakes. Proceedings of the IEEE Computer Society Conference on Computer Vision and Pattern Recognition.

[13] Xu C., Prince J.L. (1998). Snakes, shapes, and gradient vector flow. IEEE Trans. Image Process..

[14] Kass M., Witkin A., Terzopoulos D. (1988). Snakes: Active contour models. Int. J. Comput. Vis..

[15] Li B., Acton S.T. (2007). Active contour external force using vector field convolution for image segmentation. IEEE Trans. Image Process..

[16] Tang C., Lu W., Cai Y., Han L., Wang G. (2008). Nearly preprocessing-free method for skeletonization of gray-scale electronic speckle pattern interferometry fringe patterns via partial differential equations. Opt. Lett..

[17] Tang C., Ren H., Wang L., Wang Z., Han L., Gao T. (2010). Oriented couple gradient vector fields for skeletonization of gray-scale optical fringe patterns with high density. Appl. Opt..

[18] Li Y.H., Chen X.J., Qu S.L., Luo Z.Y. (2011). Algorithm for skeletonization of gray-scale optical fringe patterns with high density. Opt. Eng..

[19] Jiang W., Ren T., Fu Q. (2024). Deep learning in the phase extraction of electronic speckle pattern interferometry. Electronics.

[20] Feng S., Chen Q., Gu G., Tao T., Zhang L., Hu Y., Yin W., Zuo C. (2019). Fringe pattern analysis using deep learning. Adv. Photonics.

[21] Liu C., Tang C., Xu M., Hao F., Lei Z. (2020). Skeleton extraction and inpainting from poor, broken ESPI fringe with an M-net convolutional neural network. Appl. Opt..

[22] Lychev S., Digilov A., Djuzhev N. (2024). Galerkin-Type Solution of the Föppl–von Kármán Equations for Square Plates. Symmetry.

[23] Eichhorn N., Osten W. (1988). An algorithm for the fast derivation of line structures from interferograms. J. Mod. Opt..

[24] Ciarlet P.G. (2025). Linear and Nonlinear Functional Analysis with Applications.

[25] Mittelstedt C. (2023). Theory of Plates and Shells.

[26] Goodman J.W. (2007). Speckle Phenomena in Optics: Theory and Applications.

[27] Dainty J.C. (2013). Laser Speckle and Related Phenomena.

[28] Wang Q., Li W., Bao W., Zhang F. (2022). Accelerated randomized coordinate descent for solving linear systems. Mathematics.

[29] Lychev S., Digilov A., Demin G., Gusev E., Kushnarev I., Djuzhev N., Bespalov V. (2024). Deformations of Single-Crystal Silicon Circular Plate: Theory and Experiment. Symmetry.

[30] Bychkov P.S., Lychev S.A., Bout D.K. (2019). Experimental technique for determining the evolution of the bending shape of thin substrate by the copper electrocrystallization in areas of complex shapes. Vestn. Samara Univ. Nat. Sci. Ser..

